# Land reversion and zoonotic spillover risk

**DOI:** 10.1098/rsos.220582

**Published:** 2022-06-08

**Authors:** John E. Vinson, Nicole L. Gottdenker, Luis Fernando Chaves, RajReni B. Kaul, Andrew M. Kramer, John M. Drake, Richard J. Hall

**Affiliations:** ^1^ Odum School of Ecology, University of Georgia, Athens, GA 30602, USA; ^2^ Center for the Ecology of Infectious Diseases, University of Georgia, Athens, GA 30602, USA; ^3^ Department of Veterinary Pathology, College of Veterinary Medicine, University of Georgia, Athens, GA 30602, USA; ^4^ Department of Infectious Diseases, College of Veterinary Medicine, University of Georgia, Athens, GA 30602, USA; ^5^ Instituto Conmemorativo Gorgas de Estudios de la Salud, Apartado Postal 0816-15 02593, Panamá, República de Panamá; ^6^ Department of Integrative Biology, University of South Florida, Tampa, FL 33620, USA

**Keywords:** zoonotic spillover, land reversion, land-use change, disease management

## Abstract

Deforestation alters wildlife communities and modifies human–wildlife interactions, often increasing zoonotic spillover potential. When deforested land reverts to forest, species composition differences between primary and regenerating (secondary) forest could alter spillover risk trajectory. We develop a mathematical model of land-use change, where habitats differ in their relative spillover risk, to understand how land reversion influences spillover risk. We apply this framework to scenarios where spillover risk is higher in deforested land than mature forest, reflecting higher relative abundance of highly competent species and/or increased human–wildlife encounters, and where regenerating forest has either very low or high spillover risk. We find the forest regeneration rate, the spillover risk of regenerating forest relative to deforested land, and how rapidly regenerating forest regains attributes of mature forest determine landscape-level spillover risk. When regenerating forest has a much lower spillover risk than deforested land, reversion lowers cumulative spillover risk, but instaneous spillover risk peaks earlier. However, when spillover risk is high in regenerating and cleared habitats, landscape-level spillover risk remains high, especially when cleared land is rapidly abandoned then slowly regenerates to mature forest. These results suggest that proactive wildlife management and awareness of human exposure risk in regenerating forests could be important tools for spillover mitigation.

## 1. Introduction

A major contributor to human disease risk is human-induced land-use change, particularly deforestation [1–5]. Associated changes in habitat suitability for wildlife can influence animal and plant community structure and species interactions, including host–parasite interactions [6,7], potentially leading to pathogen spillover from wildlife to humans [6,8,9]. Deforestation may lead to community disassembly, with highly competent species remaining [10–13], increase infection susceptibility in remaining species via stress-mediated effects [14–16], alter host behaviour and movement [17,18] and/or create niches for competent vectors [19,20]. Mosaic land-use and monoculture agriculture can create suitable conditions for zoonotic spillover via contacts between wildlife, domesticated animals and people [17,18,21–24]. Spillover risk may subsequently decline under urbanization as habitats become inadequate for wildlife reservoirs [25].

Once deforested, if the land is no longer agricultural or further developed, different post-disturbance reforestation regimes, including commercial tree plantations, non-assisted regeneration or directed regeneration/rehabilitation, could influence spillover risk in different ways [23,26]. If wildlife communities rapidly reassemble in reforesting landscapes, reservoir hosts that thrive in human-dominated landscapes may decline in relative abundance, reducing transmission potential for zoonotic pathogens such as Lyme [27] and Chagas disease [11]. Alternatively, slow revegetation of regenerating land could maintain canopy gaps used by edge-adapted vectors, or prevent recolonization by keystone predators and long-lived, low-competence species, maintaining relatively high transmission potential in regenerating habitats [28,29]. Since reforestation can provide benefits for integrated social-environmental systems, it is crucial to identify conditions under which land reversion augments spillover risk to inform appropriate interventions.

Our goal here is to develop initial theory on the effects of land reversion on spillover risk, using a simple mathematical model of land-use change. Although deforestation–spillover relationships can be context-dependent [30], we assume that deforested habitat poses the greatest spillover risk to humans [5,31,32], and explore two scenarios where regenerating land has either low or high spillover risk. We investigate how land reversion influences transient dynamics and cumulative spillover risk for each land-use change scenario.

## 2. Methods

**Figure 1.**
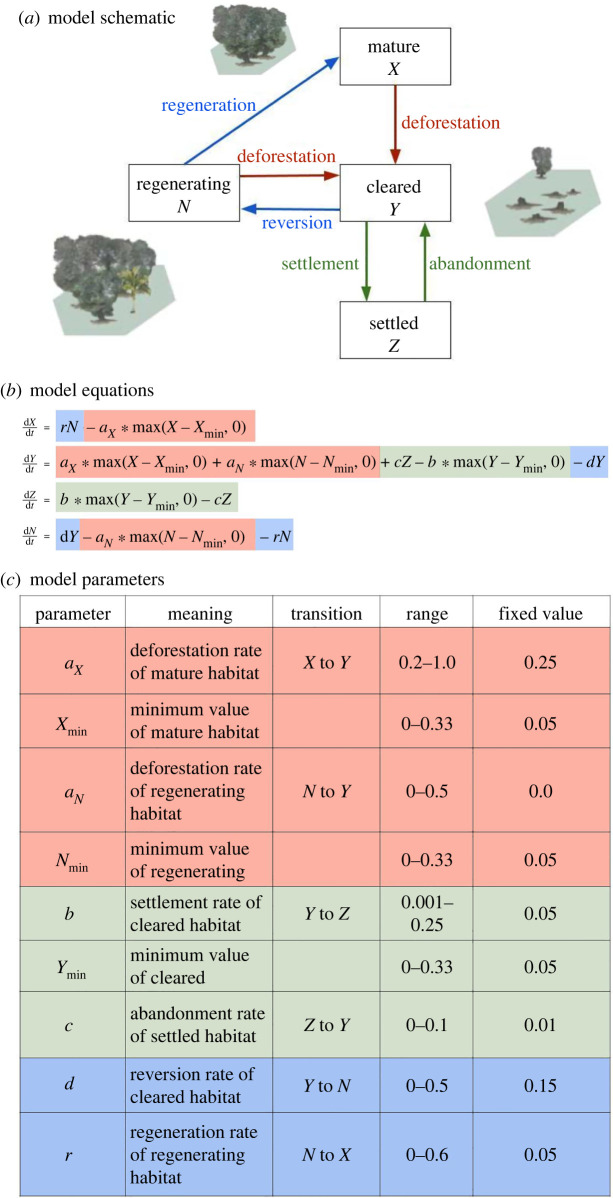
(*a*) Compartmental model describing the dynamics of land conversion and reversion. Each box represents a habitat type and arrows represent land transition processes. Red arrows represent the deforestation of mature or regenerating habitat to cleared land. Blue arrows represent the reversion of cleared land to regenerating forest, and subsequent regeneration to mature forest. Green arrows represent the creation or abandonment of settled habitat. The corresponding differential equations (*b*) and model parameters (*c*) are colour-coded to match the transition processes in the conceptual diagram. The parameter table defines each parameter, its default value, and the range used in sensitivity analyses (electronic supplementary material, figure S1). All transition rates between habitats are assumed to be proportional to the amount of land being converted; additionally, deforestation and settlement rates are assumed to decline to zero once a minimum area of each habitat type remains, reflecting economic or physical constraints on land conversion.

Our model comprises a system of differential equations that describe transitions between the proportions of four land-use types (figure 1). Mature habitat (*X*) is late-successional, contiguous forest, with high species richness and diverse ecological interactions that repress spillover risk. Cleared habitat (*Y*) is land partially or completely deforested for human use, ranging from pasture with remnant shade trees to large-scale monoculture. We assume that cleared habitat has high spillover risk relative to mature forest. This could reflect ecological mechanisms where wildlife reservoirs reach high abundance through loss of forest-specialized competitors and predators, or competitive dominance of edge or human-adapted reservoirs (such as rodents [33] or mosquito vectors [34]). Additionally, this habitat may have higher risk reflecting higher encounter likelihood between wildlife reservoirs, domestic animals and people. Settled habitat (*Z*) represents areas where many natural landscape features have been removed, such as urban areas; although these areas have high human density, we assume that there is low relative abundance of wildlife reservoirs and bridging domestic hosts, resulting in a low spillover risk. Regenerating habitat (*N*) is created when cleared habitat reverts (or is proactively restored) toward mature successional forest; aside from a few well-studied multi-host zoonotic pathogens, such as the bacteria responsible for Lyme disease [27] relatively few empirical studies have documented the relative abundance of wildlife reservoirs and human usage of regenerating habitat compared with mature and cleared habitats. However, structural differences in tree height and canopy complexity, light and relative humidity and accessibility and use frequency by humans could result in regenerating habitat being more similar to cleared habitat or mature habitat, or distinct from other habitats, in its relative spillover risk.

We assume that each habitat type *i* ∈ {*X, Y, Z, N*} has a habitat-specific probability of a zoonotic spillover event occurring per unit area per unit time, *p_i_*(*t*), which could depend on transmission ecology (e.g. abundance of wildlife hosts/vectors capable of transmitting to humans), human population density and socio-economic and cultural factors shaping human–wildlife contacts. We define the landscape-level spillover risk, *p(t)*, as the sum of the proportion of each habitat type at time *t* multiplied by its habitat-specific spillover risk: $p(t)=\sum_{i}^{\left\{X,Y,Z,N\right\}}\;p_{i}(t)\times i(t).$

To focus on the contributions of land reversion on spillover risk, we initially make the strong assumption that habitat-specific spillover probabilities per unit area are constant, i.e. they are independent of the total amount of each habitat, and of changing human density, across the timescale of deforestation. Because this ignores reductions in spillover risk resulting from low initial human density, or reductions in local wildlife reservoir abundance due to fragmentation effects, this assumption reflects a ‘worst-case’ scenario for spillover risk, where high local wildlife reservoir densities and/or human use of risky habitats result in a high annual spillover probability. We further assume that spillover risk is much higher in cleared habitat than mature forest and settled habitat (i.e. $p_{X},p_{Z}\approx 0$), such that the probability of at least one spillover event occurring per year if the entire landscape was cleared is very high (i.e. $p_{Y}\approx 1$).

We explore two scenarios for the spillover risk of regenerating habitat: (i) spillover risk in regenerating habitat is very low $\left(\;p_{N}\approx 0\right)$, which could occur if reservoir hosts and vectors decline rapidly during regeneration, or human use of secondary forest is low; and (ii) spillover risk is high in regenerating habitat, comparable to the spillover risk in cleared habitats ($p_{N}=p_{Y}\approx 1$), which could occur if wildlife reservoirs maintain high abundance during forest regeneration. Under these scenarios, landscape-level spillover risk is approximately equal to (i) the proportion of cleared habitat (*p = Y*) or (ii) the proportion of cleared and regenerating habitats (*p = Y + N*), respectively. Additionally, we relax the assumption of constant habitiat-specific spillover risk to explore these two scenarios when local spillover probabilities are time-varying due to positive local reservoir density-habitat amount relationships (as could occur under fragmentation) and where human use of risky habitats scales with the amount of settled habitat (as a coarse proxy for human population density) (electronic supplemental material, S1 and S2). To explore the effect of land history on spillover risk, we assumed two initial landscape configurations: (i) mostly mature habitat (i.e. prior to extensive deforestation), and (ii) a mixed-habitat mosaic (75% cleared: 25% mature habitat).

We quantify the short-term, long-term and cumulative response of landscape-level spillover risk (*p*) to land-use change with the following metrics: short-term spillover risk is quantified by the peak value of spillover risk following the onset of deforestation, and the timing of the peak; long-term spillover risk by the equilibrium value, and cumulative landscape-level spillover risk as the integral of the instantaneous spillover risk over 100 years, divided by the maximum possible spillover risk during this period (=100). We chose 100 years as a plausible upper limit over which our functional forms for deforestation or regeneration rates are applicable. We compared these metrics of spillover risk for our two scenarios about the relative spillover risk of regenerating habitat to a baseline scenario where no land reversion occurs (i.e deforestation is a one-way process). To assess the generality of our results, and to identify the key land-use change rates determining spillover risk, we conducted a sensitivity analysis where we covaried land-use change parameters and recorded the net effect of each parameter on cumulative landscape-level spillover risk using partial rank correlation coefficients (electronic supplementary material, S3; default parameter values and ranges listed in figure 1*c*).

## 3. Results

### 3.1. Spillover risk through time

**Figure 2.**
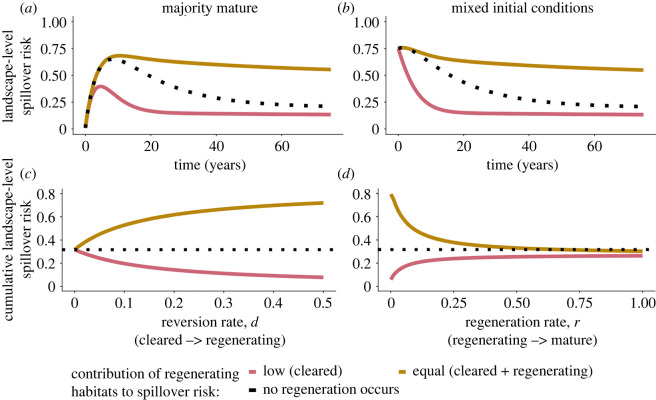
The influence of land history and relative risk of regenerating habitat on landscape-level spillover risk. Spillover risk dynamics through time when (*a*) landscape is initially dominated by mature forest or (*b*) landscape is a mosaic of 75% cleared: 25% mature. (*c*,*d*) Cumulative landscape-level spillover risk (i.e. area under the spillover curve for 100 years) as functions of (*c*) the reversion rate of cleared to regenerating habitat and (*d*) the regeneration rate to mature habitat. Line colour represents two scenarios for the relative spillover risk of regenerating habitat relative to cleared land: regenerating land has near-zero spillover risk (gold) or spillover risk is high in cleared and regenerating habitats (pink). The black dotted line represents a null scenario when no land reversion occurs.

We found that land-use history influences the trajectory of spillover risk through time. When the initial landscape is mostly mature forest, landscape-level spillover risk initially peaks then falls as cleared land, which has the highest spillover risk, is created and subsequently lost to urbanization and reforestation (figure 2*a*). However, if the initial landscape is mostly cleared, spillover risk is initially high then decreases (figure 2*b*). Landscapes retain high spillover risk through time when regenerating habitat has similar spillover risk to cleared habitat (figure 2*a*,*b*, gold lines). This can occur when regenerating habitats retain simplified wildlife communities similar to cleared lands. Conversely, peak spillover risk is lower, and decreases more rapidly, when regenerating habitat has low spillover risk relative to cleared habitat, e.g. when reservoir hosts aggregate around agricultural food subsidies, but rapidly disperse once these lands are abandoned (figure 2*a*,*b*, pink lines). Accounting for dynamic changes in habitat-specific spillover probabilities (due to changing local host density and human population size) tended to lower spillover risk through time, with the most pronounced effects at the onset of deforestation, when both cleared and settled habitats (and thus wildlife reservoirs and human populations) were low; electronic supplementary material, figures S1 and S2 a,b.

### 3.2. Cumulative spillover risk

We explored how cumulative landscape-level spillover risk was influenced by the rates at which regenerating habitat is created (i.e. the reversion rate from cleared habitat, *d*) or lost (i.e. the regeneration rate to mature forest, *r*). When regenerating habitat has low spillover risk, land reversion always reduces spillover risk (figure 2*c*,*d* pink). Spillover risk declines most rapidly when reversion is fast and regeneration to mature forest is slow (figure 2*d* pink). However, when regenerating and cleared habitats contribute equally to spillover risk, land reversion always increases cumulative spillover risk, especially when regeneration is slow (figure 2*c*,*d* gold). When local spillover probabilities varied with changing wildlife and human population sizes, cumulative spillover risk showed qualitatively similar relationships with reversion and regeneration rates, but was slightly lower than when local spillover probabilities were assumed constant (electronic supplementary material, figures S1 and S2c-d). When mature forest had a low but non-zero spillover risk (0.25, one quarter of the spillover risk of cleared land), the trajectories of landscape-level spillover risk through time were qualitatively similar (electronic supplementary material, figure S3).

### 3.3. Sensitivity analysis

When only cleared habitat contributed to spillover risk, the most influential parameters on cumulative spillover risk related to the removal of cleared habitat (electronic supplementary material, figure S4a). If cleared land is only developed for settlement when the total amount of cleared land is above a threshold (*Y*_min_), increasing this threshold generally results in higher spillover risk because more high-risk cleared habitat is retained. However, increases in settlement (*b*) and reversion (*d*) rates, which remove high-risk cleared habitat, reduce spillover risk. When both cleared and regenerating habitats contribute to spillover risk, the regeneration rate to mature forest (*r*) becomes influential and reduces spillover risk (electronic supplementary material, figure S4b). For our previous analyses, we assumed that the deforestation of regenerating, secondary forest (*a_N_*) does not occur. When only cleared habitats have a high spillover risk, increasing the rate of deforestation of secondary forests increases landscape-level spillover risk. However, when both cleared and regenerating have a high spillover risk, increasing the deforestation rate of regenerating secondary forests decreases landscape-level spillover risk. This is because when secondary forest is re-cleared, some of that cleared land is potentially converted to settled habitat, which has low relative spillover risk.

## 4. Discussion

It is well understood that deforestation can increase spillover risk by altering wildlife communities and increasing human-pathogen contacts [1–5]. However, it remains unknown whether reforestation would then reduce spillover risk, and over what timescale. Using a simple model of land-use change, our goal was to develop baseline theoretical predictions for how land reversion influences spillover risk, to motivate future empirical and modelling research that layers in the interactive effects of landscape structure, transmission ecology and human behaviour. We found that land reversion can influence short- and long-term spillover risk in opposing ways, depending on attributes of regenerating land (e.g. wildlife community composition) and the rates at which it is created or lost. The greatest landscape-level spillover risk occurs when regenerating and cleared habitats have high spillover risk, and secondary forest slowly regains attributes of mature forest. This pattern was robust to variation in land-use change parameters, and still held when land-use dynamics influenced local spillover probabilities (via hypothesized effects on wildlife and human populations).

Recent work suggests that restoring natural habitats could improve ‘landscape immunity’ by reducing wildlife aggregation in cleared habitats [35]. Whether this approach could work crucially depends on whether regenerating forest retains high spillover risk. We might expect regenerating forests to retain high spillover risk when host communities remain simple, e.g. due to a lack of forest complexity, simplified food webs or because potentially diluting species slowly recolonize [36,37]. Additionally, if environmental conditions remain similar to cleared habitat (e.g. canopy gaps retain abiotic features of open habitats), this could favour edge-adapted, human-associated species. Alternatively, regenerating forest may enhance landscape immunity if adjacent mature forest allows recolonization of species that dilute the relative abundance of reservoirs, or if reservoir hosts attracted to cleared land by agricultural food subsidies disperse once this subsidy disappears.

While few empirical studies have explored the effects of regenerating habitats in contributing to spillover risk, several studies have found an increase in parasite transmission in reforesting areas. For example, Morand & Lajaunie [38] found that reforestation can contribute to disease outbreaks when mature forest is cleared for production of tree plantations, such as oil palm [38]. Some countries where forest expansion is occurring through land abandonment, active reforestation and afforestation have shown a positive relationship between reforestation and parasite transmission [39]. For instance, host communities in recently reforested areas in the United States and Italy caused tick populations to increase dramatically, increasing the incidence of tick-borne disease in humans [28,29], while tropical secondary forests host elevated populations and infection of triatomine vectors of Chagas disease [40]. While our model includes the processes of reversion and regeneration, empirical studies should identify and specify the type of habitat reversion that is occurring (e.g. abandonment versus plantation and the scale and rate of these processes), and determine the relative spillover risk of these habitats.

Our preliminary exploration of effects of habitat fragmentation and human use on reducing spillover risk have implications for mitigating zoonotic pathogen spillover via the promotion of land reversion/regeneration or reducing human exposure in risky regenerating areas. Small-scale, traditional agroecological practices and the development of high-quality agricultural matrices may help mitigate spillover events from occurring by encouraging highly diverse species communities in reforesting habitats [41], attracting pollinators and seed dispersers that enhance forest regeneration [42] and attracting wildlife that may be hunted sustainably [22,43–45]. Applying social science approaches that investigate activities of people who use forests to understand awareness of transmission in ‘risky’ habitats is critical to developing disease mitigation strategies [46]. Similarly, it is necessary to investigate structural factors related to the political economy driving the emergence of pathogens in dynamic landscapes [47–50].

### 4.1. Model assumptions, limitations, and future directions

While our model has provided insight into how reversion can influence spillover risk on a changing landscape, it has some strict simplifying assumptions and limitations. Spillover events can occur following changes in human-wildlife contacts, wildlife community competence, and human behaviors, all of which are influenced by changes in the landscape. In our analyses, we left the specific ecological and/or socio-economic mechanisms by which spillover can increase or decrease following land change events undefined. Initial exploration that included proxies for fragmentation effects and increasing human density through time suggest that our general results are robust. However, future work could explicitly incorporate information on habitat amount-reservoir abundance relationships and habitat-specific human exposure into our framework to generate more quantitative predictions for a focal system (e.g. Chagas or Leishmaniasis).

This study showed that the relative spillover risk in regenerating habitat is a critical determinant of landscape-level spillover risk. Therefore, transdisciplinary studies are urgently needed to quantify how sylvatic transmission, human-use and behaviour, and human–wildlife contacts differ between primary and secondary forest. Within each habitat, metrics that quantify seasonal changes in transmission potential for the ecological communities, such as indices of relative abundance of competent hosts and vectors, and infection prevalence in hosts or vectors that can transmit to humans, could reveal within-year patterns of spillover risk. In addition to measuring the differences in human use of primary and secondary forests, more detailed studies are needed of how human behaviour results in pathogen exposure in these habitats, as well as the socio-economic drivers of the creation and loss of secondary forests.

Our study provides a foundation for future modelling work that incorporates additional biological and social realism into processes influencing land-use change and zoonotic spillover. By linking host and vector abundance to the amount and distribution of each habitat type, future work could further integrate the dynamics of the land-use model with a traditional parasite transmission model to quantify habitat-specific transmission potential, and in turn quantify human cultural and behavioural processes that determine exposure [51].

## Data Availability

The research uses no data. R code used for simulation and to generate figure 2 can be accessed at https://doi.org/10.6084/m9.figshare.c.5578710. The electronic supplementary material can be accessed at https://doi.org/10.6084/m9.figshare.c.6002301 [52].
